# Open Air Treatment

**Published:** 1878-09

**Authors:** 


OPEN AIR TREATMENT.—The "open air" treatment of wounds is meeting with its advocates. It is said to be extensively in vogue in Russia where dressings are dispensed with and the air allowed immediate contact. It is claimed by the advocates of this plan that Lister's treatment is useful only in that it renders innoxious the pernicious material of dressing, and that the plan of freedom from dressings and open contact with the air is followed by better results than any other.—*Mich. Med. News.*

